# Serological and molecular prevalences and phylogenetic analysis of *Coxiella burnetii* in dogs in Al-Qadisiyah and Baghdad Provinces, Iraq

**DOI:** 10.14202/vetworld.2024.2603-2611

**Published:** 2024-11-22

**Authors:** Hadaf Mahdi Kadhim, Mithal K. A. Al-Hassani, Ahlam Ali Soghi Al-Galebi, Israa M. Essa

**Affiliations:** 1 Department of Biology, College of Education, University of Al-Qadisiyah, Al-Diwaniyah, Iraq; 2 Department of Public Health, College of Veterinary Medicine, University of Basrah, Basra, Iraq

**Keywords:** canine zoonotic diseases, *Coxiellosis*, polymerase chain reaction, Q-fever, sequencing analysis

## Abstract

**Background and Aim:**

*Coxiella burnetii* is a highly contagious zoonotic bacterial micro-organism. This study aimed to estimate the prevalence of *C. burnetii* in dogs using serological and molecular methods. Furthermore, a sequencing analysis of *C. burnetii* dog isolates was conducted.

**Materials and Methods:**

A total of 172 dogs, including 93 pet dogs, 21 police dogs, 38 guardian dogs, and 20 stray dogs, were selected. Venous blood was drained from the dogs and examined serologically by indirect enzyme-linked immunosorbent assay (ELISA) and molecularly by polymerase chain reaction (PCR) for *C. burnetii*. A sequencing analysis of *C. burnetii* dog isolates was conducted.

**Results:**

The overall prevalence of *C. burnetii* was 16.86%, accounting for 55% in stray dogs, 9.68% in pet dogs, 19.05% in police dogs, and 13.16% in guardian dogs. Strong positive sera were observed in stray dogs (4.84 ± 0.29), whereas weak sera were observed in pet dogs (3.22 ± 0.18). PCR analysis revealed 6.4% positive dogs, accounting for 1.08%, 4.76%, 2.63%, and 40% in pet, police, guardian, and stray dogs, respectively. Phylogenetic tree analysis of local *C. burnetii* isolates revealed a total rate of similarity and mutations/changes between 95.47% and 100% and 0.059%, respectively. Subsequently, the local isolates were significantly similar to Chinese hedgehog, Iraqi camel, and Colombian human *C. burnetii* National Center for Biotechnology Information-GenBank isolates.

**Conclusion:**

This is the first study on prevalence of *C. burnetii* in dogs in Iraq. To prevent transmission of *C. burnetii* to humans, the role of dogs or other domestic and wild animals as sources of infection must be investigated extensively. In addition, the prevalence of *C. burnetii* in other Iraqi regions should be surveyed using the most sensitive and specific diagnostic assays, such as ELISA and PCR.

## Introduction

*Coxiella burnetii* is an obligate intracellular, pleomorphic, Gram-negative, spore-forming, coccobacillary bacterium that belongs to the *Coxiellaceae* family, the *Legionella* order, and the *Gammaproteobacteria* phylum [1, 2]. This bacterium was first observed and isolated in Australia and the USA from 1920 to 1935. Although the morphological characteristics of *C. burnetii* are similar to those of *Rickettsia*, it exhi- bits some physiological and genetic variations [3–6]. This bacterium can also affect domestic and wild animals and humans, causing a disease known as Q fever or coxiellosis, which is endemic to several countries worldwide [7]. After infection, a large amount of *Coxiella* is excreted *via* milk, urine, feces, placenta, aborted fetuses, or reproductive tissues and then transmitted directly or indirectly to other animals or humans [8]. The inhalation of airborne materials, especially after animal birth, is a common cause of human infection [9]. In addition to mice and birds, more than 40 species of ticks have been found to play a role in the transmission of infection from animal to animal and from animal to human [7, 10]. Like intracellular micro-organisms, the acute phase produces antibodies that are provided by cellular response; in the chronic phase, high antibodies lead to the formation of immune complexes [6, 11, 12].

In dogs, *C. burnetii* infection is primarily characterized by the lack of symptoms or the presence of non-specific clinical symptoms, including depression, lethargy, seizures, and fever until the disease has progressed, thereby causing reproductive problems, such as stillbirth and deformities [13, 14]. Despite the development of a simple and reliable medium for screening *C. burnetii*, this bacterium remains an infectious organism [11, 15, 16]. Thus, enzyme-linked immunosorbent assay (ELISA), a sensitive, specific, ready-to-use, and commercially available diagnostic method, can be used to identify specific antibodies [17]. However, the correlation between seropositivity to *C. burnetii* and bacterial shedding and clinical diseases in dogs remains unclear, and whether an increase in seroprevalence increases the risk of infection in dog owners remains unknown [18]. In recent decades, polymerase chain reaction (PCR), a safe, highly sensitive, specific, and easy-to-perform laboratory diagnostic tool, has been primarily used to identify the acute phase of *C. burnetii* infection [19, 20]. However, the combination of PCR and serology is recommended for the definitive diagnosis of early and late stages of *C. burnetii* infection [21].

No studies have been conducted in Iraq to identify *C. burnetii* in dogs; therefore, this study aimed to estimate the prevalence of *C. burnetii* in dogs serologically by ELISA and molecularly by PCR. Furthermore, a sequencing analysis of *C. burnetii* dog isolates was conducted.

## Materials and Methods

### Ethical approval and Informed consent

This study was approved by the Scientific Committee of the Department of Biology (College of Education, University of Al-Qadisiyah, Approval No. 121/CVM-UQ/18-10-2023). Blood samples were collected after verbal consent from all animal owners.

### Study period and location

This study was conducted from November 2023 to February 2024 among various regions in Al-Qadisiyah and Baghdad Provinces (Iraq), and the samples were tested at the Microbiology Laboratory in the College of Veterinary Medicine (University of Al-Qadisiyah).

### Samples

Samples were collected using convenience sampling. A total of 172 dogs, including 93 pets, 21 police dogs, 38 guardians, and 20 strays, were selected. Under aseptic conditions, approximately 4 mL of venous blood was collected from the cephalic or saphenous veins of each study dog and then transferred into two labeled tubes: 2.5 mL of the collected venous blood was placed in an ethylenediaminetetraacetic acid -anticoagulant tube (Falcon, Jordan) and frozen at −20°C until molecular examination and 1.5 mL of the collected blood was placed in a free-anticoagulant glass-gel tube. After centrifugation (2800 × *g* for 5 min), the obtained sera were placed in labeled 1.5-mL Eppendorf tubes (Abdos, India) and frozen at −20°C until tested serologically.

### Serological analysis

The kit contents were prepared, and the sera were thawed in accordance with the manufacturer’s instructions for the indirect canine anti-Q-fever antibody ELISA kit (Biotangusa, USA). Then, the positive and negative solutions and the serum samples were diluted and processed. In addition, the optical density (OD) was measured at 450 nm using an automatic ELISA microplate reader (Bio Tek, USA). Positive anti-Q-Ab samples were identified after determining the P/N value (OD _samples_/OD _negative control_) at ≥2.1, whereas samples with a P/N value of <2.1 were considered negative.

### Molecular testing

In the present study, a Presto Mini gDNA Bacteria kit (Geneaid, Taiwan) was used to extract DNA from whole blood samples from the study dogs. After estimating the concentration and purity of DNA using a nanodrop spectrophotometer (Thermo Scientific, UK), a GoTaq Green Master Mix kit (Promega, Korea) was used to prepare the Master Mix tubes by targeting the *16S rRNA* gene ([F: 5′-AGT ACG GCC GCA AGG TTA AA-3′] and R: [5′-CTC CAA TCC GGA CTA CGA GC-3′]) at a final volume of 20 μL [22]. Using a thermal cycler (Bio-Rad, USA), PCR was performed as follows: 1 cycle for initial denaturation (95°C/5 min); 30 cycles for denaturation (95°C/40 s), annealing (56°C/40 s), and extension (72°C/40 s); and 1 cycle for final extension (72°C/7 min). The electrophoresis of PCR products in agarose gel (1.5%) stained with ethidium bromide was performed at 100 V and 80 A for 1 h. Based on the standardized band sizes of a ladder marker, the product size of positive samples was identified at approximately 425 bp under an ultra-violet illuminator (Clinx Science, China).

### Sequencing analysis

Positive PCR samples (n = 11) were sequenced using the modified Sanger method (Macrogen Company, Korea), and the received data of local *C. burnetii* strains were subjected to multiple sequence alignment analysis and phylogenetic tree analysis using MEGA 11 software (Pennsylvania State University, USA) to investigate their identity for National Center for Biotechnology Information (NCBI)-Basic Local Alignment Search Too *C. burnetii* isolates. Furthermore, the local *C. burnetii* strains were submitted to the NCBI database under the following GenBank IDs: PQ097649.1, PQ097650.1, PQ097651.1, PQ097652.1, PQ097653.1, PQ097654.1, PQ097655.1, PQ097656.1, PQ097657.1, PQ097658.1, and PQ097659.1.

### Statistical analysis

All obtained results were documented in Microsoft Excel 2010 (Microsoft Office, Washington, USA) and statistically analyzed using Student’s unpaired t-test and one-way analysis of variance in GraphPad Prism software version 9.4.1 (GraphPad Software Inc, USA). Differences between the compared values were considered significant at p < 0.05 (*), p < 0.01 (**), p < 0.001 (***), and p < 0.0001 (****) [23]. Values are represented as either mean ± standard error or percentage (%).

## Results

**Figure-1:**
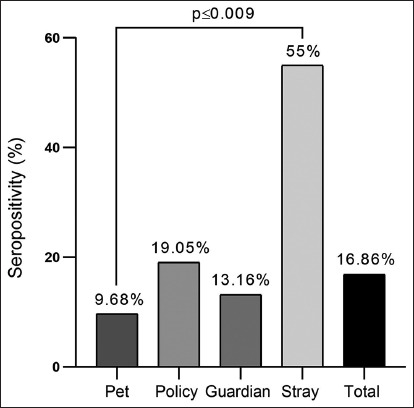
Seropositive results of enzyme-linked immunosorbent assay in study dogs (n = 172).

**Figure-2:**
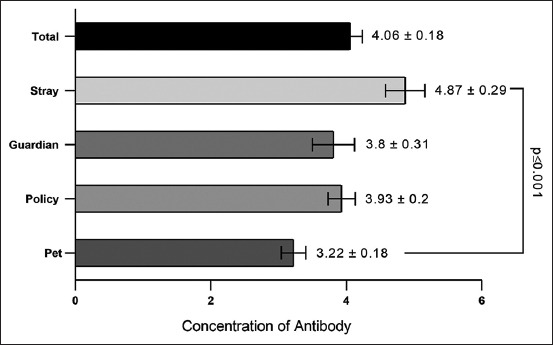
Concentration of antibody values in the seropositive study dogs (n = 172).

The overall *C. burnetii* serological prevalence was 16.86% (n = 29) among the 172 dogs tested. In addition, the prevalence of *C. burnetii* infection among the study dogs was significantly different (p ≤ 0.009), accounting for 55% (n = 11) in 20 stray dogs, 9.68% (n = 9) in 93 pet dogs, 19.05% (n = 4) in 21 police dogs, and 13.16% (n = 5) in 38 guardian dogs (Figure-1). Significantly, the antibody concentration was higher in stray dogs (4.87 ± 0.29) and lower in pet dogs (3.22 ± 0.18) compared with guardian (3.8 ± 0.13) and police dogs (3.93 ± 0.2) (Figure-2).

**Figure-3:**
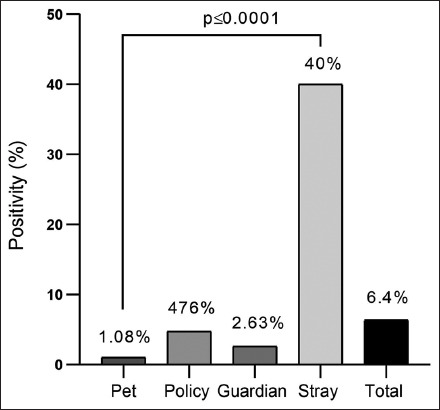
Polymerase chain reaction results in the study dogs (n = 172).

Based on the PCR test results, among 172 dogs, 6.4% (n = 11) were positive for *C. burnetii*, including 1.08% (n = 1) from 93 pet dogs, 4.76% (n = 1) from 21 police dogs, 2.63% (n = 1) from 38 guardian dogs, and 40% (n = 8) from 20 stray dogs (Figure-3).

**Figure-4:**
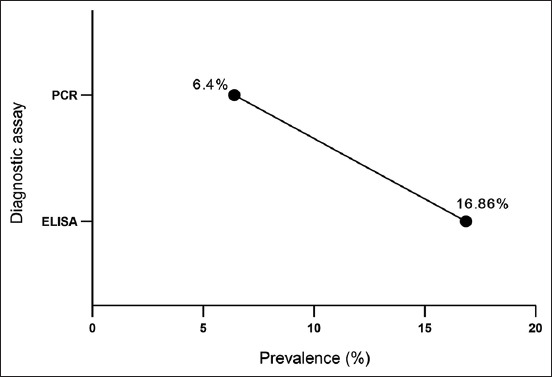
Enzyme-linked immunosorbent and polymerase chain reaction assay results.

The results of the present study indicated that the serological and molecular prevalence rates of *C. burnetii* in dogs were 16.86% and 6.4%, respectively (Figure-4).

**Figure-5:**
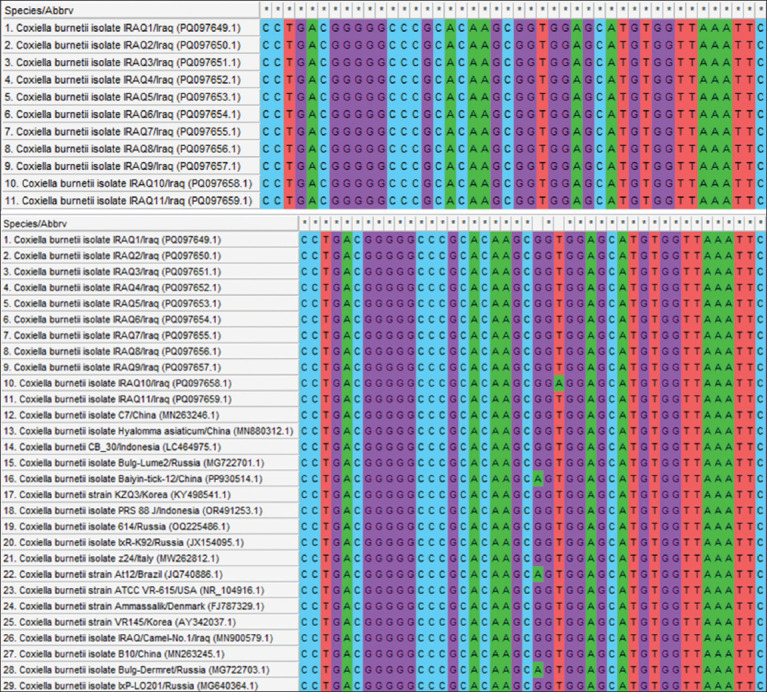
Multiple sequence alignment analysis of the local study *Coxiella burnetii* isolates and National Center for Biotechnology Information GenBank *C. burnetii* isolates/strains using MEGA 11 software.

**Figure-6:**
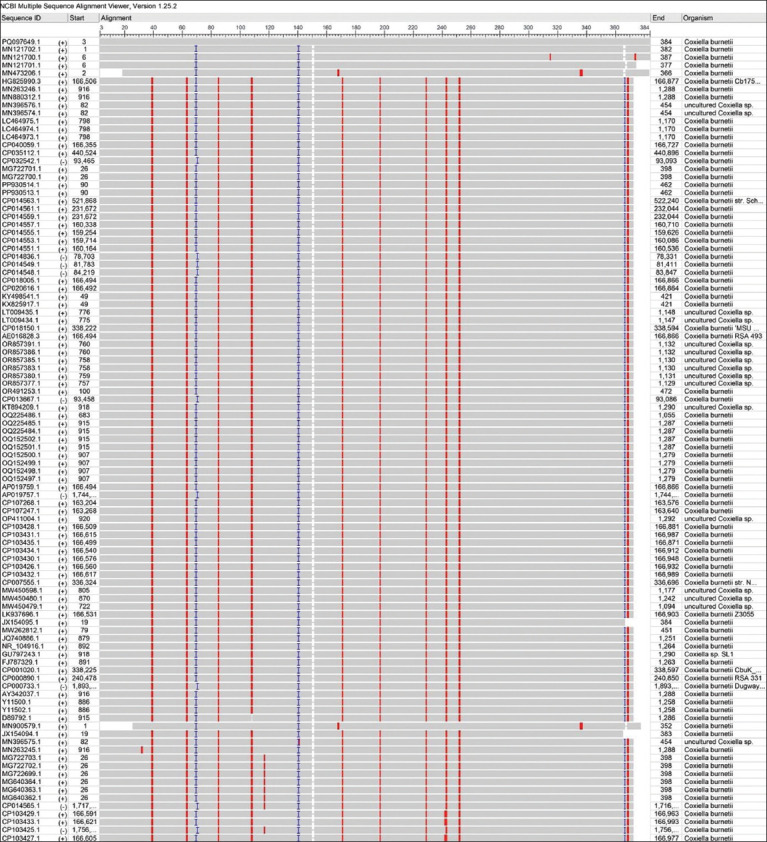
Multiple sequence alignment analysis of *Coxiella burnetii* isolates and the National Center for Biotechnology Information -GenBank *C. burnetii* isolates/strains.

**Figure-7:**
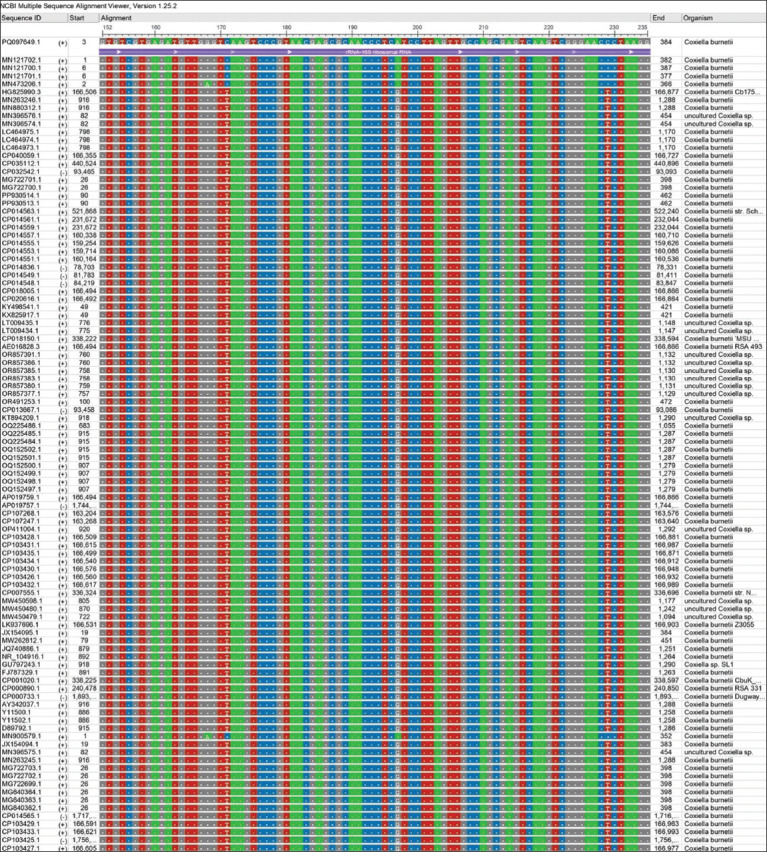
Color coded of the local study *Coxiella burnetii* isolates and National Center for Biotechnology Information -GenBank *C. burnetii* isolates/strains using the National Center for Biotechnology Information viewer showing nucleotide similarity (*) and mutations.

Sequencing data of all positive PCR products of *C. burnetii* (11) were subjected to multiple sequence alignment analysis using MEGA 11 software (Figure-5) and the NCBI viewer to detect nucleotide similarity (*) and mutation/changes between local *C. burnetii* isolates and NCBI-GenBank *C. burnetii* isolates/strains (Figures-6 and 7).

**Figure-8:**
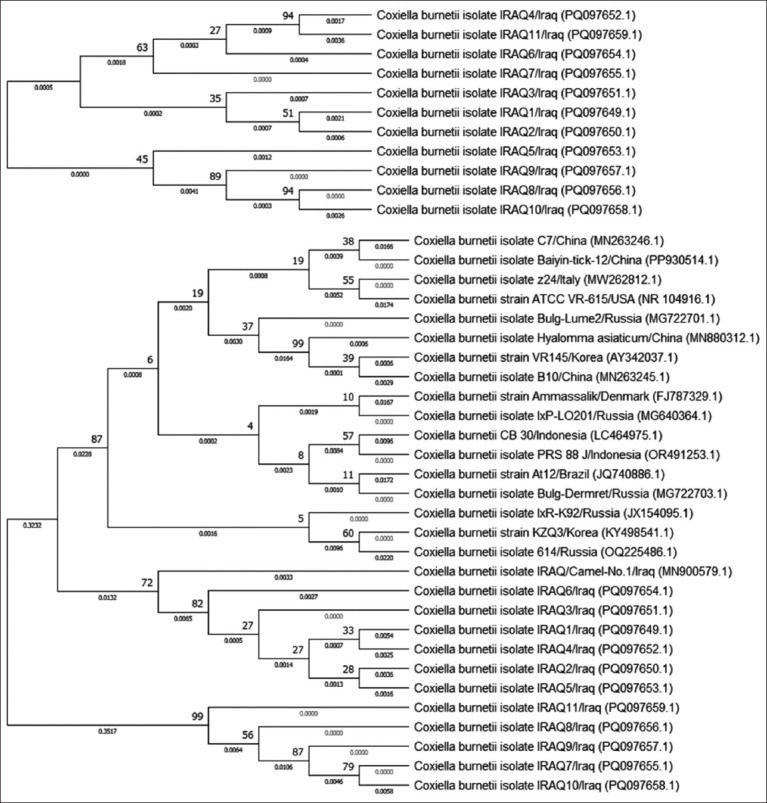
Phylogenetic tree analysis of local *Coxiella burnetii* strains and the National Center for Biotechnology Information-GenBank *C. burnetii* isolates/strains.

Comparative identification of the genetic variation between local *C. burnetii* isolates and NCBI-GenBank *C. burnetii* isolates/strains revealed that the total similarity ranged from 95.47% to 100%, whereas the total mutation rate/change was 0.059%. Subsequently, the following local isolates showed significant identity with different NCBI-GenBank isolates/strains: IRAQ1 isolate (PQ097649.1) from the Chinese hedgehog isolate (MN263246.1); IRAQ2-IRAQ6 (PQ097650.1, PQ097651.1, PQ097652.1, PQ097653.1, and PQ097654.1) from the Iraqi camel isolate (MN900579.1); and IRAQ7-IRAQ11 (PQ097655.1, PQ097656.1, PQ097657.1, PQ097658.1, and PQ097659.1) from the Colombian human isolate (MN540441.1) (Table-1 and Figure-8).

**Table-1:** Homology sequence analysis identity between the local *Coxiella burnetii* strains and the NCBI-GenBank *C. burnetii* isolates/strains.

Local strains		NCBI-GenBank			Identity (%)
	
Name	Accession No.	Accession No.	Country	Host	
IRAQ1	PQ097649.1	MN263246.1	China	Hedgehog	95.47
IRAQ2	PQ097650.1	MN900579.1	Iraq	Camel	98.03
IRAQ3	PQ097651.1	MN900579.1	Iraq	Camel	98.03
IRAQ4	PQ097652.1	MN900579.1	Iraq	Camel	98.87
IRAQ5	PQ097653.1	MN900579.1	Iraq	Camel	98.87
IRAQ6	PQ097654.1	MN900579.1	Iraq	Camel	98.87
IRAQ7	PQ097655.1	MN540441.1	Colombia	Human	100
IRAQ8	PQ097656.1	MN540441.1	Colombia	Human	99.43
IRAQ9	PQ097657.1	MN540441.1	Colombia	Human	99.45
IRAQ10	PQ097658.1	MN540441.1	Colombia	Human	99.46
IRAQ11	PQ097659.1	MN540441.1	Colombia	Human	99.45

## Discussion

In the present study, the overall prevalence of *C. burnetii* was 16.86%, accounting for 55% in stray dogs, 9.68% in pet dogs, 19.05% in police dogs, and 13.16% in guardian dogs. However, the mechanism by which dogs can maintain, transmit, and spread *C. burnetii* infection remains unclear, and differences in serological prevalence might occur throughout particular times and geographic regions. Previously, the serological prevalence of *coxiellosis* was 14.3% in stray dogs in India [24], 66.1% in stray dogs in the USA [25], 0.9% in household dogs in Italy [26], 15.0% in stray dogs in Japan [27], and 11.8% in stray dogs of Croatia [28]. In a study conducted on military army dogs, the seropositivity rates were 5.2% in French Guyana, 8.3% in Ivory Coast, 9.8% in Southern France, and 11.6% in Senegal [29]. In pet dogs, *C. burnetii* was identified in 1.9%, 2.3%, 3%, 6.5%, and 21.8% of shelters, breeding, households, aboriginal, and owned dogs, respectively [30, 31].

In the vicinity of a 2015 human Q-fever outbreak in New South Wales (Australia), Ma *et al*. [18] recorded a 26.1% serological prevalence of the bacterium in 330 dogs, indicating the role of environments in sharing infections between dogs and humans. In Portugal, Anastácio *et al*. [32] detected different positive infections in dogs in two periods (12.6% in 2012 and 1.7% in 2021), indicating the role of the sampling scheme in reducing positivity. In Brazil, 30% of police K-9 dogs were positive for anti-*C. burnetii* antibodies, indicating that working dogs can rest at a police battalion kennel and share operational incursions at a work location [33]. In Iraq, several stray dogs are found in rural areas; therefore, the apparent seropositivity of *C. burnetii* infection in stray dogs may be due to the environment and increasing direct contact with potential infectious sources. Other environmental factors include proximity to livestock transport routes and major interstate sources. Dust from trucks on animal transportation may also play a role in transmitting infections.

Higher anti-*C. burnetii* antibody concentrations in stray dogs are expected as most animals remain free, thereby increasing the chance of frequent exposure to ticks and micro-organisms through contact with wildlife and domestic animals.

The molecular PCR results revealed that 6.4% of the study dogs were positive for *C. burnetii*, accounting for 1.08% in pet dogs, 4.76% in police dogs, 2.63% in guardian dogs, and 40% in stray dogs. Globally, several studies have shown that serological tests cannot always diagnose *C. burnetii* in animals due to the absence of specific anti-*C. burnetii* antibodies at the early stage of infection [34, 35]. Comparatively, molecular examination of whole blood samples revealed a lack of Q-fever in 10% of Zambian stray dogs [36], 2.7% in different dog breeds of the Campania region in southern Italy [37], 10% in pound dogs of Algeria [35], and 11% in Iran [38]. Recently, the absence of *C. burnetii* DNA in dogs’ blood samples was attributed to bacterial shedding by dogs and cats, even in pet animals that have a higher rate of exposure to infection and breed frequently [18, 32].

According to Angelakis and Raoult [39], diseased dogs might serve as a potential source of infection to other animals during delivery and to their owners by inhaling air. Small and large ruminants, including goats, sheep, cattle, and camels, can shed large numbers of organisms during abortions and normal parturition. Moreover, considerable environmental stability can allow the persistence of *C. burnetii* in soil for a long period [22, 40].

The present study showed that 16.86% and 6.4% of the study dogs were positive for *C. burnetii* based on the ELISA and PCR results, respectively. In southern Italy, 5.97% and 2.7% of dogs were found to be positive for *C. burnetii* based on the results of ELISA and PCR, respectively [37]. Other studies demonstrated high levels of positive anti-*C. burnetii* antibodies in other domestic animals compared with the findings of molecular assays [3, 40-43]. Gangoliya *et al*. [41] detected that the overall serological and molecular positivity rates of Q-fever in sheep were 38.2% and 7.3%, respectively, whereas such rates were 23.7% and 0% in goats, respectively. ELISA and PCR showed that the positivity rates of Q-fever in one-humped camels were 19.8% and 4.4%, respectively [42]. An epidemiological study revealed that *C. burnetii* antibodies and DNA in cattle were 37% and 9%, respectively [43]. Ghaoui *et al*. [35] also mentioned that the laboratory detection of *C. burnetii* must be based on the interpretation of the serological response and presence of the pathogen. Furthermore, PCR can identify infected animals when positive serological findings are found in a herd.

Targeting the *16S rRNA* gene, phylogenetic analysis revealed that local *C. burnetii* isolates are significantly similar to Chinese hedgehog, Iraqi camel, and Colombian human isolates. Based on these data, different *C. burnetii* strains could infect dogs in Iraq, with the possible transmission of *C. burnetii* infection from different animals to humans and vice versa. Chitanga *et al*. [36] assumed that *C. burnetii* has a sylvatic and domestic transmission cycle in which livestock plays an important link between the two cycles.

## Conclusion

The overall prevalence of *C. burnetii* in dogs was high, particularly in stray dogs that showed an exceptional increase in infection rates compared with pet and police dogs, indicating that stray dogs in Iraq are frequently exposed to sources of infection over time. In addition, local *C. burnetii* isolates are considered the first Iraqi dog isolates submitted to the NCBI-GenBank, in which the results demonstrated an association between *C. burnetii* isolates and different strains, indicating that *16S rRNA* sequencing can be used as a simple, unambiguous, transferrable genotyping approach to differentiate *C. burnetii* strains. The role of dogs or other domestic and wild animals as sources of infection must be widely initiated to prevent the transmission of *C. burnetii* infection from micro-organisms to humans. In addition, the prevalence of bacteria in other Iraqi regions should be surveyed using the most sensitive and specific diagnostic assays, such as ELISA and PCR.

## Authors’ Contributions

HMK: Molecular examination of whole blood samples by PCR. MKAA: Serological examination of sera by ELISA. AASA: Collection of blood samples, preparation of sera, and statistical analysis. IME: NCBI submission and phylogenetic analysis of *C. burnetii* isolates. All authors have read and approved the final manuscript.
